# The Role of Exosomes in Viral Hepatitis and Its Associated Liver Diseases

**DOI:** 10.3389/fmed.2021.782485

**Published:** 2021-11-22

**Authors:** Hao Zhou, Zhi-han Yan, Yuan Yuan, Chen Xing, Nan Jiang

**Affiliations:** ^1^Affiliated Hangzhou Chest Hospital, Zhejiang University School of Medicine, Hangzhou, China; ^2^Department of Hepatology, Wuxi Fifth People's Hospital, Wuxi, China; ^3^Department of Oncology, The Second People's Hospital of Yancheng City, Yancheng, China; ^4^Department of Urology, People's Hospital of Dongtai City, Dongtai, China

**Keywords:** biomarkers, exosomes, fibrosis, hepatocellular carcinoma (HCC), therapeutic targets, viral hepatitis

## Abstract

Exosomes, the important carriers between cells, can carry proteins, micro ribonucleic acids (miRNAs), long non-coding RNAs (lncRNAs) and other molecules to mediate cellular information transduction. They also play an important role in the pathogenesis, prognosis and treatment of viral hepatitis and its associated liver diseases. Several studies have reported that viral hepatitis and its associated liver diseases, including hepatitis A, B, C and E; hepatic fibrosis and hepatocellular carcinoma, were closely associated with exosomes. Exploring the role of exosomes in viral hepatitis and associated liver diseases will enhance our understanding of these diseases. Therefore, this review mainly summarised the role of exosomes in viral hepatitis and its associated liver diseases to identify new strategies for liver diseases in clinical practise.

## Introduction

Exosomes, firstly discovered in 1980, are circular or elliptical membrane vesicles of endocytic origin with a diameter of ~30–150 nm, which are released into the extracellular environment after the fusion of the polycystins and plasma membranes (1). Exosomes are secreted by different body cells, including fat, dendritic, T, B, stem and tumour cells, which could be found in blood, urine and cerebrospinal fluid (2–5). Exosomes are produced by plasmacytoid dendritic cells (pDCs) and released after the fusion of multivesicular bodies (MVB) with the plasma membrane. They are composed of proteins, peptides, lipids, messenger ribonucleic acids (mRNAs), microRNAs (miRNAs), deoxyribonucleic acid (DNA) and other components (6) and can be transported to adjacent or distant organs and tissues through blood circulation (7). They can participate in various important physiological and pathological processes of the human body and affect disease development, which plays an important role in cell communication, migration, angiogenesis, immune response and tumour cell growth (8, 9).

**Figure 1:**
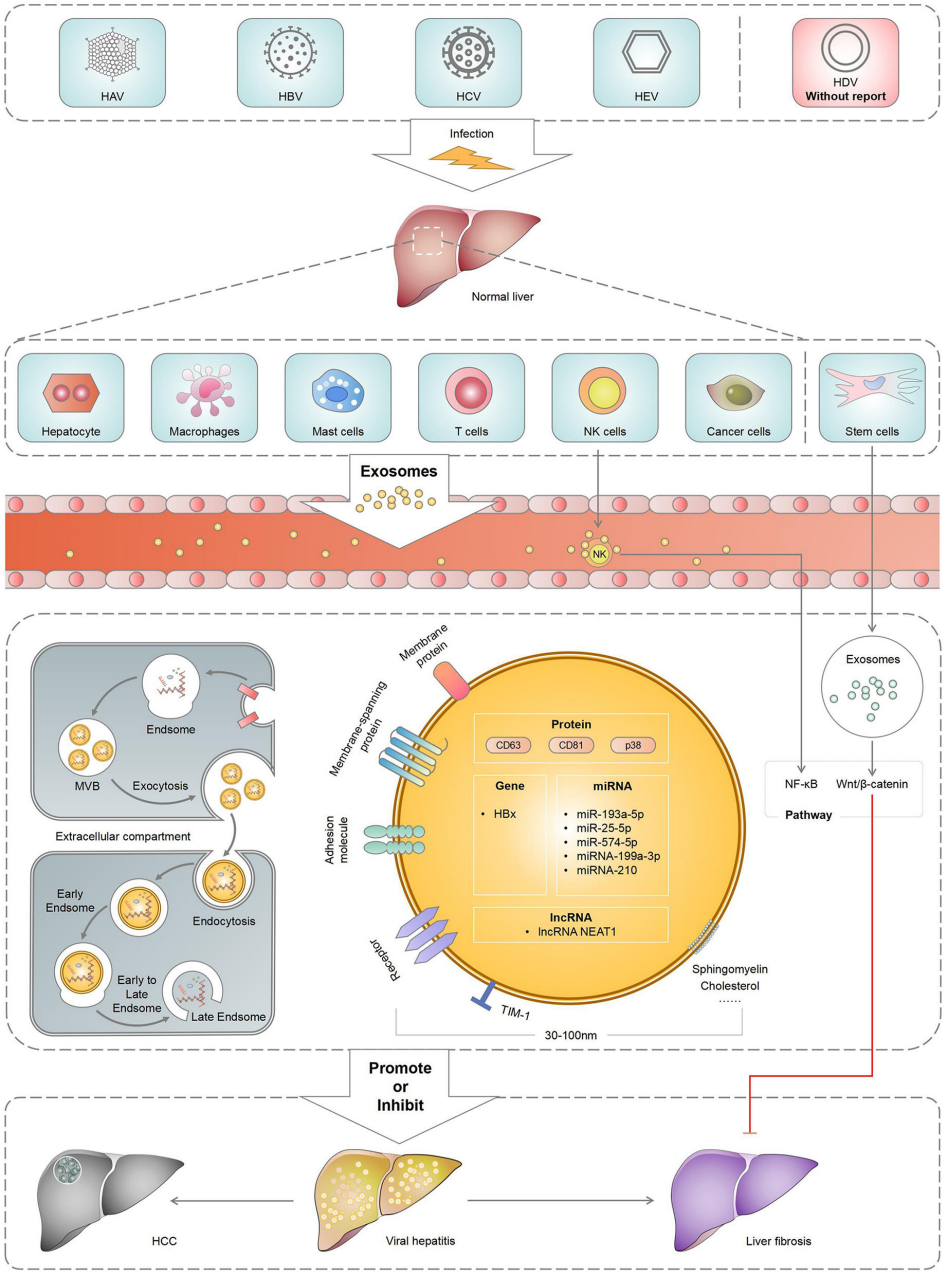
The role of Exosomes in the occurrence, development and metastasis of HCC.

Viral hepatitis occurs worldwide, including the following five viruses as the main clinical manifestations: hepatitis A, B, C, D and E. By regulating host immune response and mediating hepatitis virus replication, exosomes could influence the pathogenesis of hepatitis virus. The exosomes released from cells infected with hepatitis virus can carry nucleic and protein components, which would help hepatitis virus participate in immune escape. Meanwhile, exosomes derived from immune cells help eliminate viruses and antiviral immune defence. Exosomes released or received by the liver cells can be used for cell-to-cell communication between healthy and damaged livers (10). Moreover, exosomes produced by hepatocytes infected with the hepatitis virus spread the infection and disrupt the innate immune response of chronic viral hepatitis (11). Similarly, they can also activate the body's immune response to hepatitis infection (12). As important carriers between cells, exosomes are involved in virus transmission, immune regulation, antiviral response and viral microenvironment and play an important role in the pathogenesis, prognosis and treatment of viral hepatitis and its associated liver diseases (Figure 1). Therefore, this review mainly summarised the role of exosomes in viral hepatitis and its associated liver diseases, aiming at providing new strategies for the clinical treatment of liver diseases.

### The Role of Exosomes in Hepatitis A

Hepatitis A is caused by the hepatitis A virus (HAV) and is mainly transmitted through the faecal–oral route. HAV is a hepatophilic positive-chain RNA virus (13). Its worldwide spread is episodic and can cause acute liver disease but does not establish a persistent infection. Infected human cells can produce two types of HAV particles: non- and quasi-enveloped. Non-enveloped virus particles are stable in the faeces of infected people, whereas quasi-enveloped virus particles are present in the blood of infected individuals. The presence of quasi-envelopes protects the virus from immune response. Therefore, quasi-enveloped virus particles can spread to the liver (14).

Quasi-enveloped HAV (eHAV) was reportedly responsible for viral transmission and pDC activation (15). eHAV can be germinated from the endosomes of the HAV capsid into the MVB through the exosomes (16). HAV cell receptor 1 and cholesterol transporter NPC1 participate in the transport of exosomes from HAV-infected cells through mesh protein-mediated endogenous action, thereby promoting HAV infection (17). Furthermore, Costafreda et al. demonstrated that exosomes and HAV have similar fusion mechanisms independent of envelope glycoproteins (18). Jiang et al. confirmed that HAV structural protein pX could interact with apoptosis-associated gene 2-interacting protein X to promote virion and exogenous protein secretions through exosome-like vesicles (19).

Exosomes can protect virions from antibody-mediated neutralisation in HAV-infected cells. The presence of these exosomes can also prevent the detection of HAV by the host immune system and facilitate the spread of HAV in the liver. However, HAV virions coated with exosomes may limit replication after an eHAV infection, slowing the spread of HAV in the cells (15).

Nowadays, the diagnosis and therapeutic effects of exosomes in HAV infection have not been thoroughly explored. These results suggest that exosomes make a great difference in HAV transmission and protect HAV from the detection of the host immune system. Therefore, future studies should focus on the mechanism of action of exosomes in innate immunity and immune evasion to advance the exosomal diagnostic process and HAV infection treatment.

### The Role of Exosomes in Hepatitis B

Several studies have reported on the role of exosomes in hepatitis B (Table 1). Hepatitis B virus (HBV) is a common liver-specific enveloped DNA virus that can cause chronic hepatitis B (CHB). CHB is a global epidemic infecting ~2 billion people, of which 240 million had chronic infection (30). Approximately 650,000 people die from HBV infection and liver diseases caused by HBV infection annually (31). In recent years, several studies have demonstrated that exosomes can play a role in and influence the replication, transmission, diagnosis and treatment of HBV by regulating HBV replication and transmission. A previous study showed that immune-related miRNAs could be involved in inflammatory and immune responses (32). Zhang et al. reported that miR-199a-3p and miR-210 effectively reduced the expression of hepatitis B surface antigens (HBsAg), thus inhibiting HBV replication (20). Ninomiya et al. reported that exosome-associated tetramine CD63 contributes to the efficient assembly of HBV and its infectivity (21).

**Table 1:** The role of exosomes in hepatitis B.

**Exosome and related molecules**	**Role**	**References**
MiR-199a-3p and miR-210	The up-regulation of miR-199a-3p and miR-210 may play a role in regulating HBV replication.	(20)
Exosome-associated tetramine CD63	CD63 contributes to the efficient assembly of HBV and its infectiousness.	(21)
Exosomes derived from serum samples in CHB patients	Exosomes can induce active HBV infection and inhibit the lethality of NK cells.	(22)
Neutral sphingomyelinase2, CD9 and CD81	The HBx gene of hepatitis B virus can influence hepatic microenvironment via exosomes.	(23)
Exosomes purified from HBV-infected patients' sera	Specific proteins of serum exosomes can be used as markers of HBV and HBV-related liver cancer.	(24)
Exosomes with CD63 and albumin	Exosomes with CD63 and albumin may be early warning markers for ACLF patients.	(25)
Serum exosomal lncRNA NEAT1	The serum exosomal lncRNA NEAT1 might be a prognostic biomarker for 90-day mortality of ACLF.	(26)
Macrophage-derived exosomes	Exosomes can transfer IFN-α-related miRNAs, which can inhibit HBV replication and expression.	(27)
Exosomes from LNPCs	Exosomes can mediate the cell-to-cell transmission of IFN-α-induced antiviral activities.	(28)
LNPC-derived exosomes	Exosomes could transmit IFN-α-induced antiviral activity to HBV replicating hepatocytes	(29)

*miR, microRNA; HBV, hepatitis B virus; CHB, chronic hepatitis B; NK, natural killer; ACLF, acute-on-chronic liver failure; IFN, type I interferon; NEAT1, nuclear-enriched abundant transcript 1; LNPC, liver nonparenchymal cells*.

Similarly, exosomes can regulate immune response, revealing the underlying mechanisms of immune escape. Yang et al. analysed the serum samples of patients with CHB and found that serum exosomes contained HBV components. These exosomes can induce active HBV infection in the primitive liver cells, inhibit the lethality of natural killer (NK) cells and destroy the body's immune response, thereby promoting HBV replication and transmission (22). Kapoor et al. also found that transcription and translation products of the HBx gene in HBV can be transported to the recipient cells through exosomes and promote HBV transmission by improving the liver microenvironment (23).

Exosomes are important in the predictive diagnosis of HBV infection. Zhao et al. compared the protein composition of Huh7 cell exosomes infected with HBx and that of the control group and confirmed the presence of liver cancer-related proteins, indicating that specific proteins of serum exosomes can be considered as HBV and HBV-related liver cancer markers (24). Jiao et al. also demonstrated that exosomes with albumin and vascular endothelial growth factor (VEGF) may be more accurate and specific biomarkers for assessing liver regeneration and prognosis in patients with acute-on-chronic liver failure (ACLF), whereas exosomes with CD63 and albumin may be early warning markers for patients with ACLF (25). The serum exosomal long-chain non-coding RNA nuclear-rich transcript 1 was reported to predict the 90-day mortality in patients with ACLF (26).

Exosomes also have antiviral activity. Kwon et al. demonstrated that type I interferon-alpha (IFN-α) can be an effective treatment for HBV infection (33). Interferon can inhibit the covalent closure of circular DNAs through HBsAg and HBV, which exhibit antiviral activity and effectively inhibit HBV replication (27). Li et al. also demonstrated that antiviral response induced by IFN-α can be transported from the liver non-parenchymal cells to HBV-infected cells through exosomes, leading to the storage of immune memory and exerting antiviral functions (28). Macrophage-derived exosomes transfer IFN-α-associated miRNA from the macrophages to HBV-infected hepatocytes through endocytosis and macropinocytosis and have antiviral activity against HBV replication and expression (29).

### The Role of Exosomes in Hepatitis C

Hepatitis C virus (HCV) is a positive-chain RNA virus transmitted through the blood, affecting >71 million people worldwide (34). HCV infection is an important cause of end-stage liver disease. Therefore, the presence of exosomes is considered to play an important role in HCV replication and transmission. Masciopinto et al. reported the presence of HCV RNA after isolating exosomes from hepatocytes of patients with hepatitis C (35). Furthermore, Ramakrishnaiah et al. confirmed that HCV is transmitted by transporting exosomes between liver cells (36). HCV RNA in exosomes was also found to form protein complexes with Ago2, HSP90 and miR-122 to enhance stability and infectivity and promote replication and transmission (37).

HCV-related exosomes can play a role in the immune evasion process, and HCV can hijack exosomes released by the cells and evade the host immunity. HCV-related exosomes can also induce RUNXOR and RUNX1 expressions via the STAT3-miR124 axis, and RUNXOR and RUNX1 up-regulation may promote myeloid suppressor cell differentiation and host immune response inhibition, thereby evading host immunity (38). Ji et al. concluded that HCV can facilitate galectin-9 secretion in monocytes, which inhibit T-cell-mediated-specific immune response after interacting with T-cell Ig and mucin domain protein-3 (39).

The inhibitory effect of exosomes on viruses is considered a potential treatment for HCV infection. Exosomes can be the medium of HCV RNA transportation to pDCs (40). HCV RNAs were found to act on toll-like receptor 7 to activate pDC, thus promoting IFN synthesis and release and inhibiting HCV replication and transmission (41). Giugliano et al. found that human liver sinus endotho-othesotropic cells (HLSECs) can internalise HCV virus particles through intercellular contact, act on the conformation recognition receptor, which can up-regulate IFN gene expression, increase type I and III interferon levels, stimulate HLSECs to secrete exosomes and eventually inhibit HCV virus replication (42). Aydin et al. showed that blocking the release of extracellular vesicles and exosomes can significantly affect viral replication without affecting the host cell viability. Therefore, they suggested that inhibiting the extracellular vesicle release could be a potential antiviral strategy for the treatment of HCV and other emerging RNA viruses (43).

HCV-associated exosomes can affect virus replication and transmission and mediate immune evasion (Table 2). Further efforts are needed to explore the role of exosomes in HCV infection and to provide new ideas for the diagnosis and treatment of HCV infection.

**Table 2:** The role of exosomes in hepatitis C.

**Exosome and related molecules**	**Role**	**References**
HCV-CD81	HCV-CD81 complex may leave cell in the form of exosomes.	(35)
Hepatocyte-derived exosomes	Hepatic exosomes would transmit productive HCV infection *in vitro*.	(36)
Exosomes isolated from HCV-infected individuals or Huh7.5 cell supernatants	HCV RNA in exosomes can form protein complexes to and promote its replication and transmission.	(37)
HCV-related exosomes	HCV-related exosomes can induce RUNXOR and RUNX1 expression via the STAT3-miR124 axis.	(38)
HCV-RNA-containing exosomes	HCV RNA can be transported to plasmacytoid dendritic cells through exosomes.	(40)
Exosomes derived from HLSECs	HLSECs induce the release of antiviral exosomes to inhibit HCV replication.	(42)
Extracellular vesicles	The inhibition of extracellular vesicle release may be a potential antiviral strategy for the treatment of HCV.	(43)

*HCV, hepatitis C virus; RUNXOR, RUNX1 overlapping RNA; RUNX1, runt-related transcription factor 1; STAT3, signal transducer and activator of transcription 3; HLSEC, human liver sinusoidal endothelial cells*.

### The Role of Exosomes in Other Hepatitis Infections

To date, the role of exosomes in hepatitis D virus has not been systematically reported. However, as an intestinally transmitted and liver-obsessed virus, the effects of exosomes on hepatitis E virus (HEV) and its scoring model have been mostly investigated (44). Exosomes participate in the immune escape of HEV, enrich the cholesterol and phosphatidylserine levels, increase the HEV intake in the liver cells and promote HEV replication and transmission (45). Nagashima et al. found that HEV was transferred and released through MVB. The mechanisms underlying HEV infection in rats may be similar to those in humans (46). Primadharsini et al. confirmed that HEV in rats is released through MVB screening and that HEV release in rats requires a pathway associated with exosomes (47).

Non-enveloped HEV and eHEV enter the cells through different mechanisms. The main route of eHEV entry into the cells is through the mesh protein-mediated endophagus. Compared with non-enveloped HEV, eHEV binds to the cells much less efficiently and requires longer inoculation time to achieve its maximum infectiousness (48). Degradation of the eHEV membrane in the lysosomes to achieve membrane removal may greatly increase its infectiousness. Non-competitive neutral phospholipase inhibitors GW4869 or silent Rab27A/Hrs gene expression can inhibit the secretion of exosomes, resulting in a significant reduction in HEV release, providing a new treatment strategy for hepatitis E (46).

### The Role of Exosomes in Hepatitis-Associated Hepatic Fibrosis

Hepatic fibrosis (HF) is caused by excessive production and accumulation of insoluble collagen and extracellular matrix components after sustaining chronic liver damage. Various chronic liver diseases can lead to HF and even liver cirrhosis. Activation of hepatic stellate cells (HSCs) is a primary event that results in HF development (49).

Exosomes can promote HF development. Exosomes from damaged liver cells are rich in cytochrome P450, and the reactive oxygen produced by cytochrome P450 2E1 (CYP2E1) can produce superoxide anion free radicals, hydrogen peroxide and strong oxidants, and increased CYP2E1 levels under various pathophysiological conditions can lead to hepatocellular apoptosis through the oxidative stress mechanism (50). Exosomes from damaged hepatocytes containing cytochrome P450 are speculated to be involved in the development of fatty degeneration by increasing the expression of fibrin and hepatocyte apoptosis (51). Hepatocyte lipotoxic fatty acid damage produces exosomes rich in miR17-92 clusters, which can be absorbed by HSCs, resulting in fibrotic activation (52). Exosomes released from the epithelial cells can activate fibroblasts to trigger fibrosis. Furthermore, exosomes produced by damaged epithelial cells are absorbed by adjacent fibroblasts, resulting in increased production of α-smooth muscle actin and type I collagen to drive HF (53). Exosomes from CCL4-processed hepatocytes include different types of self-RNA and toll-like receptor 3, which can increase IL-17 production in the liver γδT cells. Increased inflammatory cytokine levels were closely associated with HSC activation (54). T cells produced by IL-17 can regulate TGF-β1 in the Kupffer cells and directly activate HSCs (55).

The expression pattern of miRNAs in the serum rich in exosomes is a highly potential biomarker for diagnosing the grade and stage of liver diseases. Niu et al. analysed the serum exosomes of patients and rats with HF and found that exosome miR-155 can serve as a non-invasive biomarker for the diagnosis and progression of HF (56). Chen et al. also showed that miR-103-3p in the serum exosomes of patients with HF may be an HF biomarker (57). Exosomes can also be used for the treatment of HF. Exosomes from healthy humans may be beneficial to patients with HF, and the primary mechanism for repairing damaged liver may be the release of paracrine factors (58). Existing reports demonstrated that exosomes are the source of umbilical cord-filled, fat-filled and bone marrow interstitial stem cells for the possible treatment of HF (59–61).

HF formation is closely associated with HSC activation. Exosomes can regulate HSC activation and have an anti-fibrosis effect. Chen et al. found that serum exosomes from healthy donors have anti-fibrosis properties, partly owing to specific miR components with therapeutic effects on activated HSCs or damaged liver cells. Serum exosomes in healthy individuals have anti-fibrosis effects. MiR-34c, miR-151-3p, miR-483-5P, miR-532-5P and miR-687 expressions were higher in healthy mice than those in mice with fibrosis, and these miRNAs can inhibit the expression of fibrogenic genes in activated HSCs (62). Exosomes derived from the human bone mesenchymal stem cells were reported to reduce HF by inhibiting Wnt/β-catenin signalling to prevent HSC activation (61). Autophagy in HSCs was reported to reduce HF by inhibiting the release of fibrotic exosomes, indicating that exosomes can serve as potential new anti-fibrosis biological agents and have a positive therapeutic effect against fibrosis and important transformational significance for the treatment of fibrosis-related diseases (63).

Exosomes can promote and antagonise HF. Activated HSCs can also release fibrin-rich exosomes, suggesting that they are new biomarkers of potential pathological conditions and play a key role in the identification and treatment of HF-related diseases.

### The Role of Exosomes in Hepatitis-Associated Hepatocellular Carcinoma

Hepatocellular carcinoma (HCC) is a highly life-threatening cancer and the leading cause of death in patients with cirrhosis. Its incidence in China accounts for 50% of global cases and deaths (64). Researchers have been focusing on the early diagnosis and treatment of HCC (65). Exosomes can functionally carry active proteins, RNA and other types of molecules that are associated with the cancer pathology (66). Therefore, investigating the role of exosomes may promote HCC diagnosis and treatment.

Exosomes can be involved in the occurrence, development and metastasis of HCC mainly through RNA transport and protein-mediated cellular communication. Kogure et al. found that exosomes in HCC cells contain varied miRNAs and can significantly promote the non-adhesive growth of liver cancer cell strains to promote tumour progression by regulating the transformational growth factor in their receptor cells to activate the kinase-1 (TAK1) signalling pathway (67). Li et al. reported that exosomes can transfer long-chain non-coding RNA FAL1 into HCC cells to promote cell growth, proliferation, migration and invasion (68). Exosomes derived from HCC cells (HepG2) were reported to be actively internalised by adipocytes, causing significant transcriptomic changes. Adipocytes treated by tumour exosomes could promote tumour growth, enhance angiogenesis and recruit more macrophages in a mouse model (69). Chen et al. also demonstrated that exosomes from highly metastatic MHCC97H cells can be ingested by the less metastatic HCC cells and subsequently promote malignant behaviours of the recipient cells. Exosomes derived from tumours may promote epithelial-to-mesenchymal transformation through signal transduction, further promoting HCC invasion and metastasis (70). Furthermore, Wei et al. found that Vps4A can regulate the secretion and ingestion of exosomes containing oncogenic and tumour suppressor miRNAs, and its down-regulated expression in HCC tissues can promote HCC development and metastasis (71). Recently, the loss of miR-320a was found to inhibit the miR-320a-PBX3-MAPK signalling pathway, induce epithelial–mesenchymal transformation and cyclin-dependent kinase-2 and MMP-2 expressions to promote the HCC development and metastasis (72).

The exchange of RNA and protein through exosomes not only plays a key role in the HCC pathogenesis and progression but also identifies specific and sensitive biomarkers for HCC recurrence and prognosis as potential non-invasive biomarkers and therapeutic targets. Examination of exosomes is conducive to promptly reflect the severity and possible progression of the disease and to control the development of the disease in the high-risk population. Serum exosomes hsa-circ-0028861 and hsa-circ-0070396 can serve as new biomarkers of HCC caused by HBV (73, 74). MiR-125b-5p and miR-223-3p can also be used as novel non-invasive biomarkers for HBV-positive HCC at an early CHB stage (75). Circulating exosome differentiation of antagonistic non-protein-coded RNAs is highly correlated with disease progression of HCV-associated HCC and may be a non-invasive prognostic biomarker for HCV-associated HCC (76).

Exosomes for the treatment of HCC are receiving increasing attention, including the adipose mesenchymal hepatocyte-, hepatocyte- and dendritic-cell-derived exosomes. Lou et al. transfected AMSC with miR-122, and the extracted adipose mesenchymal hepatocyte-derived exosomes changed the miR-122 target gene expression so that cancer cells could be sensitised to chemotherapeutic drugs. Intratumoral injection of exosomes could significantly improve the anti-tumour effects of sorafenib on HCC *in vivo* and enhance the chemotherapeutic sensitivity of HCC (77). Cheng et al. demonstrated that hepatocyte-derived exosomes could inhibit the HCC cell progression through the STAT3 pathway (78). A study that injected dendritic-cell-derived exosomes expressing AFP into the HCC mouse model found that DEXAFP was thought to induce a strong antigen-specific immune response, which significantly inhibited the HCC occurrence in mice (79). Currently, the application of exosomes in the treatment of HCC is limited to basic experiments, and further studies are required to explore the applications of exosomes in clinical practise.

## Conclusion

Exosomes can carry proteins, miRNAs, lncRNAs and other molecules to mediate cellular information transduction, which plays a bidirectional role in viral hepatitis and its associated liver diseases. They can also encapsulate and transport the hepatitis virus, promote viral replication and transmission, mediate the antiviral response and serve as the target of immunotherapy. Furthermore, exosomes can reverse fibrosis and become the key mediators of fibrosis formation. Cell communication between exosomes can promote HCC development and metastasis. However, they can also inhibit the occurrence of HCC as an immunosuppressor. The mechanisms of exosome communication will enhance our understanding of liver pathophysiology, indicating their great potential as molecular biomarkers for the diagnosis and prognosis of liver diseases and as new therapeutic methods. Although studies on exosomes have made great progress in recent years, further efforts are required to use exosomes as biomarkers for the treatment of liver diseases in clinical practise.

## Author Contributions

HZ and NJ had the idea for the article. YY and Z-hY performed the literature search and data analysis. HZ and CX drafted and critically revised the work. All authors contributed to the article and approved the submitted version.

## Conflict of Interest

The authors declare that the research was conducted in the absence of any commercial or financial relationships that could be construed as a potential conflict of interest. The reviewer DC declared a shared affiliation with several of the authors, HZ, YY, to the handling editor at the time of review.

## Publisher's Note

All claims expressed in this article are solely those of the authors and do not necessarily represent those of their affiliated organizations, or those of the publisher, the editors and the reviewers. Any product that may be evaluated in this article, or claim that may be made by its manufacturer, is not guaranteed or endorsed by the publisher.

## Abbreviations

HAV: Hepatitis A virus
HBV: Hepatitis B virus
HCV: Hepatitis C virus
HEV: Hepatitis E virus
HCC: Hepatocellular carcinoma
CHB: Chronic hepatitis B
pDCs: Plasmacytoid dendritic cells
lncRNAs: Long non-coding RNAs
miRNAs: micro ribonucleic acids
ACLF: acute-on-chronic liver failure
HSCs: hepatic stellate cells
HF: Hepatic fibrosis.
